# A Review of Japan’s Medical Care Reimbursement Programs in Primary Care from the Perspective of Social Determinants of Health

**DOI:** 10.31662/jmaj.2024-0313

**Published:** 2025-12-26

**Authors:** Hiroko Sakurai, Kemmyo Sugiyama, Kakeru Iwase, Yoshie Yuuki, Mizuki Oonaka, Motoya Maeda, Alata A. Suzuki, Katsunori Kondo, Ai Noguchi, Daisuke Nishioka, Naoki Kondo

**Affiliations:** 1 Department of Social Epidemiology, Graduate School of Medicine and School of Public Health, Kyoto University, Kyoto-shi, Kyoto, Japan; 2 Friendry Iwaizumi, Geriatric Health Services Facility, Ryokusen-kai, Iwaizumi Town, Iwate, Japan; 3 Department of International and Community Oral Health, Tohoku University Graduate School of Dentistry, Sendai, Miyagi, Japan; 4 Department of Community Health, Public Health Institute, Shiwa, Iwate, Japan; 5 Aoga‐Shima Clinic, Aogashima, Tokyo, Japan; 6 Department of Diabetes and Metabolism, Nishiyodo Hospital, Osaka-shi, Osaka, Japan; 7 Department of Nishiyodogawa Home Medical Care and Nursing Care Cooperation Consultation Support Room, Nishiyodogawa Ward Medical Association,Osaka-shi, Osaka, Japan; 8 Nishiyodo Hospital, Osaka-shi, Osaka, Japan; 9 Department of General Internal Medicine, Nishiyodo Hospital, Osaka-shi, Osaka, Japan; 10 Department of Community Building for well-being, Center for Preventive Medical Sciences, Chiba University; 11 Research Department, Institute for Health Economics and Policy, Tokyo, Japan; 12 Senboku Clinic, Osaka-shi, Osaka, Japan; 13 Department of Medical Statistics, Research & Development Center, Osaka Medical and Pharmaceutical University, Takatsuki-shi, Osaka, Japan; 14 Department of Social Impact Assessment and Evaluation, Graduate School of Medicine and School of Public Health, Kyoto University, Kyoto, Japan

**Keywords:** incentive, social determinants of health, health insurance reimbursement, health disparities, primary care, policy

## Abstract

There is increasing awareness of the need to incorporate social determinants of health (SDH) into medical practice. However, the extent to which the reimbursement system addresses SDH remains unclear. This narrative policy review aimed to evaluate the Japanese medical reimbursement system to determine whether and to what degree it incorporates assessments and actions related to SDH, with a special focus on primary care settings. We also explored the potential impacts and challenges of these programs in addressing patients’ SDH issues.

A team consisting of physicians experienced in clinics, hospitals, home care, social epidemiological research, and a community care nurse reviewed the current reimbursement system. They identified eight medical reimbursement programs for evaluation.

Two programs directly included SDH elements (“Hospitalization and Discharge Support Fee” and “Guidance in Cooperation with Mental Health Care Fee”). The two programs were introduced in 2022. It was found that SDH assessments are often optional and need more clarity in their items; few programs offer SDH assessments in outpatient and home care settings, and there is no mandate for collaboration with community supporters.

We found the Japanese reimbursement system has provisions for some programs involving SDH. However, significant challenges remain that require revision. This study offers insights and recommendations for addressing health disparities related to SDH in the future.

## Introduction

The social determinants of health (SDH) are the social factors influencing people’s health, including socioeconomic status, psychosocial factors, built environment, and social policies. The World Health Organization’s Commission on SDH (CSDH) highlights the need to quantitatively assess health inequality due to SDH and the impacts of the countermeasures, promote collaboration among diverse institutions and professions, and address inequality by improving people’s living environments through such assessments and collaborations ^(1)^. The Japan Primary Care Association (JPCA) has outlined in the “Views and Action Guidelines on Health Inequalities (Ver. 2)” the necessity of training primary care physicians (PCPs) to engage with SDH and to promote interprofessional collaboration ^(2)^.

However, the fee-for-service reimbursement system outside of hospitalizations creates structural challenges that hinder incentivizing preventive activities, including collaborative actions with non-medical organizations in the community to provide necessary social care for patients. This structure leads to difficulties linking activities addressing SDH-related risks, potentially creating disincentives for health care facilities in the long term ^(3)^. As a response, it has been proposed to use incentive grants paid to insurers for projects such as prevention, health promotion, and medical cost optimization, as well as to reimburse medical care providers for their efforts related to SDH ^(3)^.

In Japan, the Medical Service Fee System is fundamentally based on reimbursing health care institutions for their medical services according to specific criteria managed by the Ministry of Health, Labor, and Welfare. Japan’s payment system for medical services operates primarily through the Medical Service Fee Addition and Subtraction Systems (MSFASS, in Japanese: *shinryo-hoshu-seido*). MSFASS allows adjustments to the reimbursement amount based on specific conditions. The system could encourage medical practices aligned with SDH by providing financial incentives to the practices addressing patients’ SDH problems.

However, the extent to which the MSFASS considers SDH needs to be clarified. Therefore, this narrative policy review sought to identify individual reimbursement programs that potentially promote the practices addressing patients’ SDH problems. The focus was on the programs used in primary care settings, which are expected to play a vital role in early detection and response to patients’ SDH-related issues.

## Methods and Results

To understand the current situation, the research group reviewed MSFASS reimbursement programs based on their own experiences and categorized each program as ‘clearly including,’ ‘can include,’ or ‘not at all including’ SDH elements (Supplement 1). SDH elements to be considered were determined by referring to the list of SDH elements in Views and Action Guidelines on Health Inequalities (Version 2) by JPCA ^(2)^ (Supplement 2). Ethics approval was not pursued, since this study did not involve human subjects and was based on publicly available data and literature.

Eight programs were identified (Table 1). Two programs, “Hospitalization and Discharge Support Fee (*nyuutaiin-shien-kasan*)” and “Guidance in Cooperation with Mental Health Care Fee (*kokoro-no-rennkei-sidou-ryou*),” were categorized into “clearly include (Table 2).” In the “Hospitalization and Discharge Support Fee (*nyuutaiin-shien-kasan*),” there were descriptions of SDH elements in the condition, such as “Abuse” and “Young carer,” as well as “Economic deprivation.” The “Guidance in Cooperation with Mental Health Care Fee (*kokoro-no-rennkei-sidou-ryou*)” clearly mentions “Isolation” in the condition.

**Table 1.** Summary of Reviewed Reimbursement Programs.

Program name	care setting	Year^*1^	Points^*2^	Facility conditions for receiving reimbursement^*3^	General conditions for receiving reimbursement
Hospitalization and discharge support fee (*nyuutaiin-shien-kasan*)	Inpatient	2008 (2022)	700 (general)/1300 (recovery-phase)	At least 25 collaborating institutions. Face-to-face meetings (video calls allowed) between staff of collaborative institutions at least three times a year. Collaboration record with care managers. Staff will be deployed to support each ward’s admission and discharge and regional cooperation activities.	Identify patients with factors that make discharge difficult within three days of admission and conduct early interviews. Hold a multi-professional conference within seven days of admission and create a discharge support plan.
Comprehensive function evaluation fee (*sougou-teki-kinou-hyouka-kasan*)	Inpatient	2008 (2020)	50	Have at least one full-time physician or dentist who has completed appropriate training^*4^ related to comprehensive functional evaluation or has more than one year of experience in comprehensive functional evaluation.	Target: Patients aged between 40 and 65 with a disease defined in the Long-Term Care Insurance Act or those aged 65 years and over. Comprehensive evaluation^*5^ is carried out as soon as possible after the condition of the patients has stabilized. Assessment is carried out by a doctor or dentist who has completed training^*4^.
Pre-discharge visit guidance fee (*taiin-mae-houmon-sidouryou*)	Inpatient	1990 (2018)	580	None	Target: Patients scheduled to be discharged home. Visits are made to the patient’s home during hospitalization or at the time of discharge for patients who will be hospitalized for more than one month. Post-discharge care guidance is provided, taking into account the patient’s medical condition, the structure of the patient’s home, and the patient’s ability to care for himself/herself. The content of the guidance is recorded in the medical record. Public health nurses, nurses, physiotherapists, etc., under the direction of a doctor, can also provide guidance.
Joint guidance fee at discharge 1 and 2 (*taiin-ji-kyoudou-sidouryou*)	Inpatient/Home care	2006 (2018)	400	None	Target: Patients scheduled to be discharged home. The staff^*6^ of the medical institution where the patient is hospitalized and the staff^*6^ of the medical institution where the patient will be treated at home after discharge jointly provide the guidance necessary for home treatment and provide written information.
Post-discharge visit guidance fee (*taiin-go-houmon-sidouryou*)	Inpatient	2018	580	None	Target: Patients with a high level of care^*7^ The doctor, public health nurse, midwife, or nurse provides the guidance necessary for home treatment at the patient’s home or facility. If a home care nurse accompanies the patient, 20 points will be added.
Guidance in cooperation with nursing care support fee (*kaigo-sien-nado-renkei-sidou-ryou*)	Inpatient	2010	400	None	For hospitalized patients, the doctor, or relevant medical staff, in collaboration with the care support specialist who had been in charge of the patient before hospitalization, etc., provide information on care services required after discharge from the hospital, etc. The guidance is documented in the medical record, and the prepared care plan is attached to the medical record.
Specified disease medical care management fee (*tokutei-sikkan-ryouyou-kanri-ryou*)	Outpatient	1958 (1992)	225(clinic)/147(100 beds>)/87(100 beds<)	It is not available for hospitals with more than 200 beds. The points are different for clinics, hospitals with less than 100 beds, and hospitals with less than 200 beds, and the smaller the size of the medical institution, the higher the points set.	Based on the treatment plan, medical management, such as medication, exercise, and nutrition, is provided for chronic diseases specified by the Minister of Health, Labor, and Welfare, such as lifestyle-related diseases.
Guidance in cooperation with mental health care fee 1 (*kokoro-no-rennkei-sidou-ryou*)	Outpatient	2022	350	Medical institutions have established a cooperation system with psychiatry or psychosomatic medicine. The doctor providing this guidance must have received appropriate training^*8^ in suicide prevention, etc.	Patients who are not hospitalized and are judged to be at risk of exacerbation of their mental illness or in need of referral to psychiatry or psychosomatic medicine due to isolation from the community. Screening is done using the SADPersons scale, EPDS, PHQ-9, or K-6. Patients are interviewed about their life problems, which are recorded in the medical record. Provide written information to the psychiatry or psychosomatic medicine department with which the patient is collaborating.

^*1^Year in which the reimbursement program was introduced. The year of the most recent revision is shown in ( ). ^*2^Reimbursement obtained by the medical facility when the program is used; 1 point = 10 yen. ( ) are for cases where the points differ, depending on the conditions of the medical institution. For example, the points differ between general and recovery phases based on the number of hospital beds. ^*3^Facility standards are required for the reimbursement program. ^*4^At least 16 hours of training, including workshops on comprehensive functional assessment, pharmacotherapy, etc., conducted by the Japan Medical Association, the Japan Geriatrics Society, and other relevant societies. ^*5^Comprehensive evaluation of the patient’s basic daily living skills, cognitive function, motivation, etc. ^*6^Public health nurses, midwives, nurses or assistant nurses, pharmacists, dietitians, physiotherapists, occupational therapists, speech therapists, and social workers. ^*7^Patients with the criteria for independence in daily living for older people with dementia of ≥3, or those with conditions specified by the Minister of Health, Labour and Welfare. ^*8^Training in the care of suicide attempters (psychiatric emergency version) or training in the care of suicide attempters (general emergency version) hosted by the Centre for the Promotion of Suicide Prevention and Control, an incorporated association designated by the Minister of Health, Labor, and Welfare/Training in the care of suicide attempters (family doctor version) hosted by the Centre for the Promotion of Suicide Prevention and Control, an incorporated association designated by the Minister of Health, Labor, and Welfare. Training courses organized by the Japanese Association of Clinical Emergency Medicine (JACEC), etc./Training courses organized by the operators of the projects to establish base medical institutions to support suicide attempters, etc.

**Table 2.** Results of the Reviewed Programs Contain Elements Related to SDH.

Program name	Result of review^*1^	1) SDH elements included in the conditions^*2^ (SDH elements)	2) Assessment^*3^/Care setting	3) Multidisciplinary cooperation^*4^
Hospitalization and discharge support fee	A	Economic deprivation (socio-economic status), Abuse and young carer (family)	a/Inpatient	Yes (Including cooperation with the nursing and welfare sectors)
Comprehensive function evaluation fee	B	“Comprehensive evaluation of the patient’s basic ADL, cognitive function, motivation, etc.”	b/Inpatient	No
Pre-discharge visit guidance fee	B	“the patient’s medical condition, the household structure, caregiving capacity”	b/Inpatient	Yes
Joint guidance fee at discharge 1 and 2	B	N/A	N/A /Inpatient & home care	Yes
Post-discharge visit guidance fee	B	N/A	N/A /Inpatient	Yes
Guidance in cooperation with nursing care support fee	B	“based on the patient’s physical and mental condition, etc.”	b/Inpatient	Yes
Specified disease medical care management fee	C	N/A	N/A /outpatient	No
Guidance in cooperation with mental health care fee	A	Isolation (social capital), “the patient’s challenges in life”	a/outpatient	No

^*1^A: SDH elements were “clearly include;” B: “can include;” C: “not include at all.” ^*2^SDH elements listed in the condition or where it has been determined that SDH elements can be included showed in “ ”. ^*3^Assessment of patients required to use the medical program. The assessment a: “includes SDH factors;” b: “can include;” N/A: Not mentioned in the conditions. ^*4^To use the medical program, need to work with multiple professions and provide the necessary guidance for patient care. Yes: Mentioned in the conditions; No: Not mentioned in the conditions. SDH: social determinants of health.

Despite the clear statements of SDH elements to be addressed in the two programs recognized as “clearly include,” the evaluation of SDH elements was not mandatory to receive reimbursement. The “Hospitalization and Discharge Support Fee (*nyuutaiin-shien-kasan*)” can be paid if any of the 14 components that make discharge difficult are addressed. The “Guidance in Cooperation with Mental Health Care Fee (*kokoro-no-rennkei-sidou-ryou*)” needs an additional reimbursement condition for mental status assessment. If the health care provider uses a screening tool that does not include SDH elements, the condition can be met without the SDH elements.

The care settings for the five programs that we determined “can include” SDH elements were all inpatient care. Only two programs involved outpatient care.

Regarding multi-professional partnerships, the only one that mentioned collaboration with non-medical institutions, including nursing and welfare facilities, was the “Hospitalization and Discharge Support Fee (*nyuutaiin-shien-kasan*),” which included the “number of collaborating institutions” condition. With this program, nurses, public health nurses, pharmacists, physical therapists, and doctors could perform evaluations and provide patient guidance.

## Discussion

Out of the eight programs reviewed, two newly introduced programs clearly included SDH elements. This reflects a growing awareness of the challenges older patients face, who find it difficult to be discharged to their homes amid efforts to reduce the average length of hospital stay with the recent introduction of the Diagnosis Procedure Combination system. It also reflects social issues like young carers, indicating a move to clarify the target groups for support ^(4)^. The recognition may have deepened recently due to the current trends promoting seamless care from medical institutions to daily living settings, and addressing life-related challenges surrounding diseases.

As this narrative review was conducted under the premise of a primary care setting, not including the reimbursement programs of large hospitals providing advanced medical care and respective specialties. For example, the “Continuing Care and Employment Support Guidance Fee (*ryouyou-syurou-ryouritu-sidou-ryou*)” is used chiefly at designated cancer care hospitals. The “Continuing Care and Employment Support Guidance Fee (*ryouyou-syurou-ryouritu-sidou-ryou*)” allows for documentation of information sharing regarding the continuation of employment among patients, medical institutions, and industrial doctors at the patient’s workplace to promote an understanding of health considerations for the patient’s employment continuity. Other specific programs, such as “the High-Risk Pregnancy and Delivery Coordination Guidance Fee (*hairisuku-ninsanpu-rennkei-sidou-ryou*),” intend to ensure healthy pregnancy and child development for pregnant women with mental disorders or similar issues, requiring the collection and support of information on the social background, including the living environment of pregnant women, thus inherently considering, or intervening in SDH elements. Comprehensive research, including reimbursement programs at large or specialized hospitals/clinics, is necessary for future studies.

## Policy Implication

Despite these limitations, this review serves as foundational material for examining how incentive systems within primary care reimbursement can potentially promote SDH. Considering the results, we identified three challenges of the programs we reviewed in promoting the practices addressing patients’ SDH-related issues in primary care settings via the MSFASS: (1) evaluating SDH is optional, and assessment items are unclear; (2) diversification of clinical settings can assess SDH is necessary; and (3) items that encourage collaboration with non-medical and long-term care sectors are not often included. Below, we provide suggestions for overcoming each of these challenges.

(1) Evaluating SDH is optional, and assessment items need to be clarified. Clarifying the SDH elements that medical institutions need to apply for the reimbursement programs would be highly useful for promoting evaluation and related research to improve the MSFASS. Standardizing SDH’s information collection format and linking the SDH data with other patient data could improve the quality of national health care databases and progress quantitative policy evaluation using those data.

(2) Diversification of clinical settings can assess SDH is necessary. This review suggests that the setting for SDH assessment may be biased toward hospitalization. Timely assessment of SDH not only in the acute setting but also in primary care and home care settings may provide appropriate support. After the review period of this study, in 2024, the “Specific Disease Management Fee (*tokutei-shikkann-kanri-kasan*)” was revised. This revision led to the exclusion of three major diseases previously covered under this program: hypertension, dyslipidemia, and diabetes, which collectively represented 90% of the program’s coverage. These diseases are now covered under a newly established program called the “Lifestyle-related Disease Management Fee (*seikatsu-shukanbyou-kanri-ryou*).” A key requirement of this new program is that physicians must collaborate with patients to prepare a “Treatment Plan Sheet” as a condition to receive reimbursement for medical expenses. The plan could also include “other” improvement goals for work, sleep, weight loss, etc. Reviewing these items, including SDH, would help make more concrete plans.

In outpatient settings, PCPs can refer patients to their medical consultation rooms or similar services, where social workers and care managers can have coordinated discussions based the plans.

(3) Items encouraging collaboration with non-medical or long-term care sectors are not often included. To overcome this, we propose a program that encourages non-medical institutions responsible for community care and preventive care (e.g., insurers of medical/long-term care insurance) to invite medical institutions to participate in the conference on community care. For example, encouraging community comprehensive support centers, facility that collaborates with multiple professions in the community and serves as the keystone of the support network in the community, can be the program’s target. The Insurer Function Enhancement Promotion Grant and the Long-term Care Insurer Effort Support Grant for medical and long-term care insurers encourage multi-sectoral collaboration for chronic disease management and long-term care prevention ^(2)^. Within these mechanisms, there could be a proposal to further encourage collaboration with medical institutions.

One point of caution regarding implementing these proposals is that they should not cause excessive medicalization. It is crucial to realize that providing incentives for addressing social challenges, including SDH, could encroach on the benefits of welfare activities and multi-professional collaboration ^(5)^. These considerations underscore the need for a balanced approach to integrating SDH into health care practices, where incentives align with comprehensive, community-focused care strategies.

## Article Information

### Disclaimer

KATSUNORI KONDO is one of the Editors of JMA Journal and on the journal’s Editorial Staff. He was not involved in the editorial evaluation or decision to accept this article for publication at all.

### Acknowledgments

This study was supported by the Ministry of Health, Labor, and Welfare Science Research Grants, project number 21FA1012, “Research for constructing a seamless medical care system from acute to recovery and chronic phases for cardiovascular diseases,” and project number 22FA1010 “Research on identifying factors that affect the extension of healthy life expectancy and the reduction of health disparities.”

### Author Contributions

Naoki Kondo, Hiroko Sakurai, Kemmyo Sugiyama, Kakeru Iwase, Yoshie Yuuki, Mizuki Oonaka, Motoya Maeda, Alata A. Suzuki, Katsunori Kondo, and Ai Noguchi conceptualized and designed the study and searched relevant literature. Hiroko Sakurai prepared the draft. Daisuke Nishioka presented important references in the interpretation and contributed to the writing of the manuscript. All authors contributed equally to this study and approved the final version of the manuscript.

### Conflicts of Interest

None

### Approval code issued by the institutional review board (IRB) and the name of the institution(s) that approved.

Not applicable.

## Supplement

Supplementary Material
