# Supplementary material for: A Review of Japan’s Medical Care Reimbursement Programs in Primary Care from the Perspective of Social Determinants of Health
Source: JMA J. 2025 Dec 26;9(1):355–9. doi: 10.31662/jmaj.2024-0313 (PMC12889345; doi:10.31662/jmaj.2024-0313)
Supplement: Supplementary Material [file 2433-3298-9-1-0355-s001.pdf]

**Supplement 1**

**Methods**

The research group, composed of clinical professionals (physicians and nurses) with experience in hospitals, clinics, and home care in primary care settings and researchers in social epidemiology, reviewed the Medical Service Fee Addition and Subtraction Systems (MSFASS, in Japanese: *shinryo-hoshu-seido*) reimbursement programs. The authors, NK, KK, HS, Y, AS, O, and M, contributed their own expertise and clinical experience to discuss the review of MSFASS reimbursement programs via Zoom. During the discussion, when necessary, information from the Ministry of Health, Labour and Welfare (MHLW) served as the primary source regarding the proposed reimbursement programs. Reimbursement programs to be reviewed were also emailed to members who did not participate in the discussion to confirm and review the details.

Then, each research member reviewed individual programs. Extracted data covered the program name, care settings (inpatient, outpatient, home care), conditions for receiving reimbursement, SDH elements included in the conditions, assessment methods for SDH elements, and whether or not the program can promote multidisciplinary cooperation.

Feedback from other group members was compiled into a summary table to assess the extracted data. Then, we categorized each program into "clearly include," "can include," or "not include at all" SDH elements. SDH

elements to be considered were determined by referring to the list of SDH elements in Views and Action Guidelines on Health Inequalities (Ver 2) by the Japan Primary Care Association[1]. If "conditions for receiving reimbursement" included specific SDH elements in an MSFASS reimbursement program, the review members classified the program as "clearly include." Although an SDH element is not explicitly stated, the document of the program states, "screening or assessment of patients" or "collaboration with multiple professions," and all review members agreed that healthcare providers can collect information on the SDH of their patients, it was classified as "can include." When there is no reference to an SDH element in the program, it is classified as "not include at all." After categorizing all programs reviewed, review members discussed and listed potential challenges in addressing patients' SDH in the clinical setting, focusing on the clarity in the SDH elements specified in the conditions for receiving reimbursement, the measurement methods, and the simplicity of the necessary criteria for reimbursement procedure. These processes were done through the meetings held four times and continued deliberations through email. We employed the SANRA framework[2], which is a validated tool for evaluating narrative reviews to facilitate the assessment of the quality of this review (Supplement 1.1).

**References for Supplement 1**

1. Japan Primary Care Association. [Opinions and Guidelines for Action on Health Inequalities] [Internet]. Ver2. 2022 Apr 14. Available from: https://www.primary-care.or.jp/sdh/fulltext-pdf/. Japanese.

2. Baethge C, Goldbeck-Wood S, Mertens S. SANRA-a scale for the quality assessment of narrative

review articles. Res Integr Peer Rev. 2019 Mar 26;4:5.

**Supplement 1.1**

Scale for the Assessment of Narrative Review Articles – SANRA

| Item | Text from the manuscript if available | Reference section |
|---|---|---|
| Item 1: Justification of the article's importance for the readership | "Japan's payment system for medical services operates primarily through the Medical Service Fee Addition and Subtraction Systems (MSFASS, in Japanese: *shinryo-hoshu-seido*). MSFASS allows adjustments to the reimbursement amount based on specific conditions. The system could encourage medical practices aligned with SDH by providing financial incentives to the practices addressing patients' SDH problems.<br>However, the extent to which the MSFASS considers SDH needs to be clarified." | P2 L19-24 |
| Item 2: Statement of concrete aims or formulation of questions | "this policy review study sought to identify individual reimbursement programs that potentially promote the practices addressing patients' SDH problems. The focus was on the programs used in primary care settings, which are expected to play a vital role in early detection and response to patients' SDH-related issues." | P2 L24-27 |
| Item 3: Description of literature search | "The authors, NK, KK, HS, Y, AS, O, and M, contributed their own expertise and clinical experience to discuss the review of MSFASS | Supplement 1 L5-9 |

| | | |
|---|---|---|
| | reimbursement programs via Zoom. During the discussion, when necessary, information from the Ministry of Health, Labour and Welfare (MHLW) served as the primary source regarding the proposed additional reimbursement.<br>Reimbursement information was also emailed to members who did not participate in the discussion to confirm and review the details.” | |
| Item 4: Referencing | The policy implication section was developed based on the review findings (Table 2) and available literature. | P4 L25<br>Policy implication |
| Item 5: Scientific reasoning | This review was prepared based on the MSFASS. | Supplement 1<br>L5-9 |
| Item 6: Appropriate presentation of data | Table 1 showed summary of reviewed reimbursement programs.<br>Table 2 showed results of the reviewed programs contain elements related to SDH | Table 1,2 |

**Supplement 2**

**List of SDH**

Prepared by the author based on Reference 2.

| Level of SDH | |
|---|---|
| Micro | sex, age, lifestyle, socioeconomic status (education, occupation, income), gender, family |
| Meso | workplace, school environment, built environment, social capital |
| Macro | socio-political context ( economic, social and public policies), culture, climate change, natural environment, peace, social justice and equity |
